# Effect of gastroretentive gabapentin (Gralise) on postmastectomy pain syndrome: a proof-of-principle open-label study

**DOI:** 10.1097/PR9.0000000000000596

**Published:** 2017-04-11

**Authors:** Inna Belfer, Netanya I. Pollock, Jodi L. Martin, Katherine G. Lim, Carolyn De La Cruz, Gijsberta Van Londen, Stephanie R. Nunziato-Virga, Katherine Stranieri, Adam M. Brufsky, Haibin Wang

**Affiliations:** Departments of a Anesthesiology and; b Medicine, University of Pittsburgh, Pittsburgh, PA, USA

**Keywords:** Chronic pain, Postmastectomy pain syndrome, Gabapentin, Gralise, Neuropathic pain, Sensory testing

## Abstract

**Introduction:**

Chronic pain is a common and debilitating complication following breast surgery. One of the most challenging for treatment is the neuropathic pain condition, postmastectomy pain syndrome (PMPS). Gabapentin is a pharmacotherapy for neuropathic pain disorders; however, its once-daily, gastroretentive formulation, Gralise, has not been evaluated in PMPS.

**Objective:**

To evaluate the safety and effectiveness of Gralise in patients with moderate-to-severe PMPS.

**Methods:**

The primary effectiveness endpoint was a change in the worst pain intensity score from baseline to completion of 8 weeks of Gralise therapy. The secondary endpoints included the change in mood, coping behavior, sleep, and function. Sensitivity to experimental stimuli was tested before and after treatment via quantitative sensory testing. The incidence and type of adverse event were used to evaluate the safety and tolerability of Gralise.

**Results:**

Twenty-one patients with confirmed moderate-to-severe PMPS were enrolled. Nineteen of 21 (90.5%) patients completed the 8-week treatment with Gralise. A significant positive change was found in pain intensity, pain impact, and sleep. There was no change in sensory testing scores. Of total, 63.16% of patients reported reduction in present pain, 78.95% in average pain, 89.47% in worst pain, and 84.21% in overall pain severity at posttreatment visit. No significant adverse effects were noted in the study.

**Limitations:**

Variation in type of breast surgery, small sample size, lack of placebo control.

**Conclusion:**

There was a significant improvement in pain and sleep, and Gralise was well tolerated in patients with PMPS. Further investigation is warranted.

## Abstract 2

Supplemental Digital Content is Available in the Text.

An 8-week treatment with 1800 mg of Gralise decreased the severity of postmastectomy pain syndrome and the degree of sleep impairment and catastrophizing behavior.

## 1. Introduction

Breast cancer is the most common form of female cancer in the United States and worldwide.^2^ Almost half of newly diagnosed women undergo therapeutic or prophylactic breast surgery (eg, total or partial mastectomy).^26^ Among other complications from surgical treatment, chronic pain is mostly troubling, leading to suffering, disability, and reduced quality of life.^29^ The nature of pain following breast surgeries has been described as both nociceptive and neuropathic, where nociceptive pain is the sequel of damage to tissue, ligament, or muscle and can improve over time.^31^ Neuropathic pain, however, is the result of damage to and dysfunction of the nervous system and can be more debilitating.^18,25,44^ It was classified as 4 types: phantom pain, intercostobrachial neuralgia, neuroma pain (includes scar pain), and other nerve injury pain (such as motor nerve injury).^25^ Postmastectomy pain syndrome (PMPS), the most prevalent type of chronic neuropathic pain often resulting from damage to the intercostobrachial nerve irrespective of the type of mastectomy,^25^ poses treatment challenge, and improvement of treatment options is an unmet medical need.^22,43^

Gabapentin is a pharmacological option for both the prevention and relief of chronic neuropathic and postsurgical pain.^17^ It is indicated for the treatment of postherpetic neuralgia, and recent evidence suggests that perioperative use of gabapentin reduces early postoperative pain and opioid use.^32^ The inhibition of pain transmission and central sensitization with gabapentin can be explained in part by its modulating effect on calcium-induced release of glutamate from activated pain-transmitting neurons.^42^ The alternative mechanism of the antinociceptive action of gabapentin may arise through activation of noradrenergic pain-inhibiting pathways in the spinal cord and brain.^20^ The downside of pain treatment with gabapentin is the dosing regimen requiring intake 3 times a day due to its short elimination half-life and limited absorption.^42^ This regimen is associated with a high incidence of adverse effects including dizziness and somnolence, and some patients are unable to withstand the higher doses and adequate duration of treatment required for optimal pain relief.^24^

Gralise is a once-daily, gastroretentive formulation of gabapentin that gradually releases gabapentin over a 24-hour period to the optimal site of absorption, the proximal small intestine. Among other advantages, this gradual release reduces the chance of saturating intestinal uptake^10^ and is associated with an increased patient compliance and improved adverse effect profile.^11^ Due to the pain alleviation properties and enhanced tolerability of Gralise pharmacotherapy, it has been approved by the U.S. Food and Drug Administration (FDA) for patients with postherpetic neuralgia.^11^

Despite some positive evidence of PMPS relief with gabapentin,^3^ no studies have been conducted using Gralise, and its safety and efficacy profiles have not been investigated in breast cancer survivors with chronic pain. Therefore, the purpose of this study was to determine whether Gralise has a potential as a safe, tolerable, and effective treatment for PMPS and how it affects pain-related traits. In the large observational epidemiological study, we identified patients who underwent breast surgery and reported moderate-to-severe pain at 6 months or later postoperatively.^6^ We hypothesized that Gralise would reduce their pain intensity, increase sensitivity thresholds for painful stimulation, and improve mood, sleep, coping behavior, and overall pain impact scores. This study aimed to inform future randomized clinical trials with Gralise on phenotyping strategies for comprehensive evaluation of PMPS and related traits.

## 2. Methods

### 2.1. Setting and dosing

**Figure 1.**
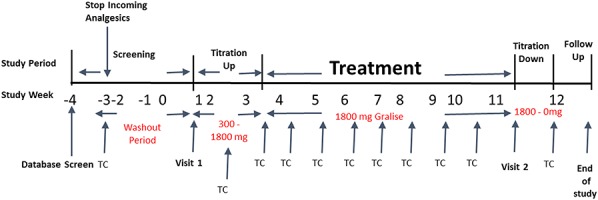
Study design. TC, telephone (survey) call.

This was a single-center, prospective, open-label, single-arm study conducted at the Clinical Research Facilities at Magee Women's Hospital of the University of Pittsburgh Medical Center in Pittsburgh, PA. The University of Pittsburgh Institutional Review Board approved the protocol, and the study was exempt of the clinical trial registry. The primary aim of the study was to assess the effectiveness and safety of Gralise in patients with moderate-to-severe PMPS. Gralise was supplied in a prepackaged form by Depomed, Inc (Newark, CA), and stored according to the manufacture's guidelines. At the first visit, subjects were dispensed a 2-week titration package, 8-week maintenance package, and 1-week discontinuing package supply of Gralise. Treatment was initiated at a dose of 300 mg/d and increased over a 2-week period to a total daily dose of 1800 mg/d. As recommended by manufacturers and previously described, 1800 mg/d is the clinically effective regimen.^11,27^ After the 2-week period, patients received a stable dose of 1800 mg/d for an additional 8 weeks. At the end of the 8 weeks, Gralise was gradually discontinued over 1 week (Fig. 1).

### 2.2. Selection criteria

The subject cohort was selected from Belfer's database of over 600 postmastectomy patients for whom pain and related psychosocial traits were previously obtained and described.^6^ In Belfer's original study, these patients were screened for postmastectomy pain at least 6 months following their surgery, and the average postoperative screening time was 38.3 months.^6^ Patients' pain quality was described using 3 instruments, The Brief Pain Inventory, The Short Form McGill Pain Questionnaire and the Breast Cancer Pain Questionnaire (BCPQ), as well as the pain burden index, as described previously, were calculated for each subject.^6^ Twenty-one patients amongst the 611 were selected to participate in this study based on the presence and scale of current postmastectomy pain, presence of neuropathic pain symptoms, overall health, history of pain management, and availability for this trial. These patients had all reported moderate-to-severe pain (Numeric Rating Scale [NRS] score ≥3/10) at the time of their initial screening and were included in this study if they reported current pain level ≥4/10 at follow-up, to ensure that their pain had not decreased spontaneously over the several year span. Medical charts were reviewed for creatinine levels, history of kidney disease, and list of pain medications. Full demographic, clinical, and medical data from eRecords were available for each patient.

### 2.3. Study patient population

Women 18 years or older who underwent total or partial unilateral mastectomy due to a breast cancer diagnosis and suffered from moderate-to-severe PMPS were eligible for the study. Moderate-to-severe PMPS was defined as shooting, pressing, burning, or pricking pain in the remaining breast, chest wall, armpit, and/or arm, persisted from the time of breast surgery and sometimes accompanied by tingling and/or numbness (as per BCPQ descriptors). Pain intensity score cutoff was ≥4/10 on the NRS, which was reported as a clinically significant level affecting patients' daily activities and quality of life.^16,28^ Patients were required to have pain level ≥4/10 at present time, which was first determined via phone interview and then confirmed at visit 1. Patients were excluded from this study if they were pregnant or breastfeeding, had any known allergy to gabapentin, had a history of gabapentin dose-limiting tolerance or lack of efficacy at doses of ≥1200 mg/d or pregabalin at doses of >300 mg/d, had any current pain management (such as treatment with duloxetine or opioids) and creatinine clearance <60 mL/L.

### 2.4. Study activities/conduct

Patients were contacted by the clinical coordinator and invited to participate in the study. There were a total of 2 clinic visits and 10 telephone visits (summarized in Supplemental Table 1, available at http://links.lww.com/PR9/A4). At the first clinic visit to the Pain Sensory Lab at the Magee Women's Hospital, confirmation of eligibility was determined via a neuropathic pain assessment by a chronic pain expert (subjects were tested for allodynia with von Frey filaments and hyperalgesia with weighted probes applied to the affected and contralateral sides as well as forearm; both phenomena are associated with neuropathic pain).^23^ All patients recruited to this study were confirmed as having PMPS with both specific pain descriptions as per BCPQ and specific sensory tests (eg, having dull pain in the primary area in response to von Frey filaments' touch and stabbing pain in response to pin prick with probe^34^). Serum creatinine levels were obtained through local laboratories. Signed informed consent was attained before study procedures from each subject. Once the quantitative sensory testing (QST) session and study questionnaires were completed during the first visit, the patient was given a Gralise supply and scheduled for a future second visit. The clinical coordinator contacted the study subject the following day to confirm that the first dose was taken. For the next 10 weeks, the patient was called weekly, and pain and adverse event (AE) data were collected via structured phone interview. If patients did not self-report drug-related side effects, patients were asked about the presence of AEs commonly observed with gabapentin treatment. During the second visit (at the end of 8 weeks of Gralise treatment), the subject completed the same surveys and underwent the same QST procedures as the first visit (baseline). A follow-up telephone call addressing any AEs was conducted at week 12 (7 days after treatment completion). The duration of the subject's participation in the study was 15 weeks (after eligibility determination), which included the 3-week washout period, 2-week titration-up period, 8-week treatment period, 1 week titration-down period, and 1 week follow-up period.

### 2.5. Pain and related trait assessment

An extensive phenotyping of pain and comorbid traits was achieved through validated questionnaires and QST (supplemental Table 1s, available at http://links.lww.com/PR9/A4). The Brief Pain Inventory (BPI)^38^ was used to characterize pain. The location, intensity (measured by the NRS), duration, frequency and quality of pain, and pain-related functional limitations were recorded. Primary endpoint was a change in the worst pain level over a 24-hour period. Anxiety, symptoms of depression, and sleep disturbances were assessed using short-form instruments from the National Institutes of Health roadmap initiative, Patient-Reported Outcome Measurement Information System (PROMIS).^7,9,12^ These instruments have been calibrated on more than 20,000 subjects and have been extensively validated in studies comparing results with established scales.^30^ Catastrophic thinking associated with pain was quantified via the Pain Catastrophizing Scale.^15,40^ This scale has been validated in patients with chronic pain.

Sensitivity to painful stimulation was assessed by measuring both mechanical (pressure) and thermal (cold pressor) pain thresholds and tolerance, as described previously.^5,34^ Pressure pain variables were determined using a digital pressure algometer (Wagner FDX, Greenwich, CT) with a flat round transducer (probe area 0.785 cm^2^) applied bilaterally at the trapezius muscle and on the dorsal aspect of the proximal forearm over the extensor muscle compartment. Pressure was increased at a steady rate of approximately 1 kg/s until the subject perceived the pressure first as painful (indicating the patient's threshold) and then unbearable (suprathreshold sensitivity or tolerance), and the pressure in kilograms was recorded.

Cold pain sensitivity was determined via immersion of the right hand in a circulating cold water bath maintained at 4°C for 30 seconds. This was repeated 3 times, with 2 minutes in between immersions. The third immersion lasted until the subject reached the pain tolerance (or a 3-minute maximum), and the time in seconds was recorded. The intensity of cold pain was rated using the NRS at the midpoint of and 30 seconds after each test. All procedures were performed by the clinical coordinator and a trained clinical volunteer under Belfer's supervision, and the questionnaires were self-administered under the supervision of the study staff. All data were entered into a web-based database in real time.

### 2.6. Statistical analysis

#### 2.6.1. Study endpoints (as predefined in the protocol)

##### 2.6.1.1. Primary

The mean change in patient reported worst pain level measured over 24 hours with NRS (via the BPI instrument) from baseline (visit 1) to week 10 (visit 2; end of treatment with 1800 mg or highest tolerated dose of Gralise).

##### 2.6.1.2. Secondary

The mean change in patient reported levels of present and average pain, mood, coping behavior, sleep, and function, as well as in pain sensitivity scores (mechanical and thermal thresholds and tolerance) from baseline to week 10.

##### 2.6.1.3. Statistical considerations

All continuous variables were normally distributed, and the mean values and SDs were calculated. To assess the change in PMPS and PMPS-related outcomes from baseline to the end of treatment, the pretreatment and posttreatment mean scores were compared using paired sample *t* tests in IBM SPSS Statistics 22.0. Differences between baseline and posttreatment values of >30% for psychosocial variables and pain sensitivity scores, and a difference of 1.5 to 2 points on the NRS for pain intensity were considered clinically significant. A probability of <0.05 was considered statistically significant. This proof-of-concept pilot study aimed to collect the data for power and sample size calculations for future larger-scale clinical trials.

## 3. Results

**Figure 2.**
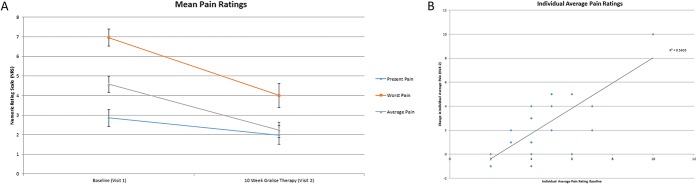
(A) Mean pain ratings from pain intensity scales. (B) Individual average pain ratings. To determine if there were subgroups of patients with different treatment responses, each subject's average pain score at baseline is compared with the change in their average pain score at visit 2.

All study participants were women of Caucasian origin. The mean age of the 21 patients was 55.2 (SD = 6.5) years old at the baseline. The mean body mass index was 27.5 (SD = 4.9). Table 1 summarized demographic data. Nineteen of 21 (90.5%) patients completed an 8-week treatment with Gralise (one patient was discontinued due to unforeseen family travel, and another one withdrew due to AEs, see page 4, paragraph 4). The subjects' mean worst pain intensity (primary outcome) decreased by 3.0 (SD = 2.4) between week 0 and week 10 (*P* = 0.0001). The subjects' average pain intensity showed a decrease of pain at a mean level of 4.6 (SD = 1.9) before treatment started to 2.2 (SD = 1.8) on the NRS at the end of 8 weeks of treatment. The mean difference of subjects' average pain intensity ratings between baseline and the end of the study was 2.4 (SD = 2.7) (*P* = 0.001). Of the 19 subjects who completed the treatment, 12 (63.2%) reported significant reduction in present pain (SD = 2.1, *P* = 0.03), 15 (79.0%) reported significantly lower average pain (SD = 2.7, *P* = 0.001), 15 (79.0%) reported significantly lower worst pain (SD = 2.4, *P* = 0.001), and 16 (84.2%) reported significantly lower overall pain severity (SD = 6.8, *P* = 0.0001) at posttreatment visit (Fig. 2A). The results from all BPI pain intensity scales are displayed in supplemental Figure 1s (available at http://links.lww.com/PR9/A4) and included in Table 2. Figure 2B shows a scatterplot with individual pain scores depicting the change in the subject's average pain rating as a function of the subject's baseline pain rating. No relationship between the 2 values was observed (*R*^2^ = 0.56).

**Table 1:**
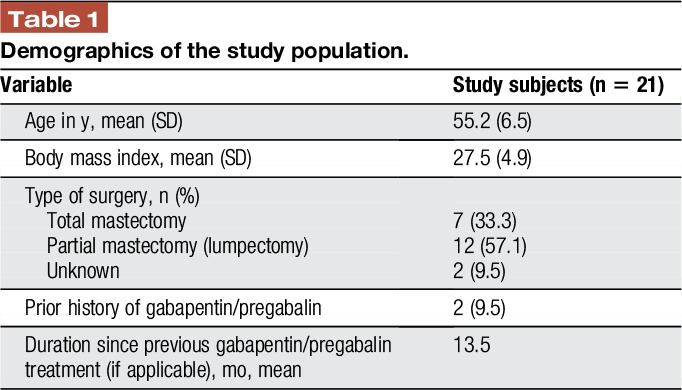
Demographics of the study population.

**Table 2:**
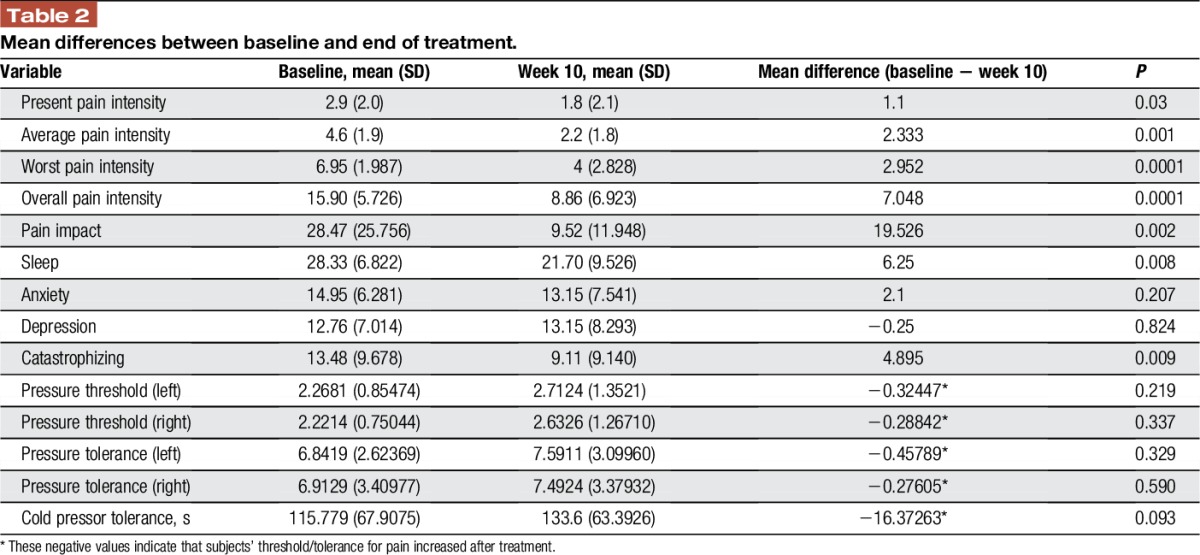
Mean differences between baseline and end of treatment.

Additional objectives for this study were to assess if there were any changes in the impact of pain on daily activities, mood, coping behavior, and sleep patterns. Compared with baseline, 73.7% of patients reported significantly lower impact of their pain on daily functioning at the posttreatment visit (SD = 23.1, *P* = 0.002) compared with their survey answers pretreatment, 38.89% of patients reported feeling less stressed, and 61.1% of patients reported having greater feelings of control. There was also a significant improvement in sleep variables after Gralise treatment compared with baseline. Of total, 61.9% of patients reported significantly improved sleep demonstrated by a decrease in restless sleep, a decrease in difficulty sleeping, and a decrease in trouble staying asleep (SD = 9.4, *P* = 0.008), while 27.8% of patients reported better sleep quality at the end of Gralise treatment, demonstrated by feeling more satisfied with sleep, feeling that sleep was more refreshing, and feeling that they got enough sleep. Posttreatment catastrophizing scores were also significantly lower (SD = 7.2, *P* = 0.009). However, Gralise was not noted to have a significant effect on anxiety or depressive symptoms (SD = 7.2, *P* = 0.2 and SD = 5.0, *P* = 0.8, respectively).

The secondary effectiveness endpoint used QST to assess if Gralise therapy improved patients' sensitivity to mechanical and thermal experimental stimulation. There was no difference between pretreatment and posttreatment scores in patients' average pressure thresholds on both the left (*P* = 0.2) and right arms (*P* = 0.3). Likewise, there was no difference in pressure threshold (*P* = 0.51) or tolerance on the left arm (*P* = 0.3) and right arm (*P* = 0.59) between pretreatment and posttreatment measurements. There was a trend towards higher tolerance (eg, less pain sensitivity) to ice-cold water when comparing baseline to posttreatment scores (*P* = 0.09). All the psychological factor data are displayed and summarized in supplemental Figure 2s (available at http://links.lww.com/PR9/A4) and Table 2.

The safety and tolerability were evaluated by the incidence of AEs reported while on Gralise therapy. No severe AEs were noted. Of the total 21 patients enrolled in the study, only 1 patient (61 years old) withdrew from the study on week 2 due to AEs (fatigue, sleeplessness, and dizziness). There were a total of 5 AEs: fatigue, dizziness, dry mouth, sleeplessness, and itchiness. All AEs were mild and did not require treatment. Fourteen patients reported at least 1 of these events. Of total, 42.9% (9/21) of the patients reported fatigue, 33.3% (7/21) reported dizziness in the morning, 4.8% (1/21) reported dry mouth, and 9.5% (2/21) reported sleeplessness. Among the 14 patients with the before mentioned side effects, 7 patients reported 2 or more. All side effects, with the exception of itchiness, subsided by week 7 of the study. One patient (4.5%) reported itchiness that lasted from week 3 to week 12. At the end of the 12 weeks, patients were asked to report their general impression of their degree of pain relief; these results were compared simultaneously with patient reports of side effects from the Gralise therapy (supplemental Figure 2S, available at http://links.lww.com/PR9/A4). Patients were categorized as having marked, mild or unchanged/worse pain relief and also none, not significant, or significant side effects. None of the patients in the study had the significant side effects of diarrhea, vomiting, nausea, or weight gain that are reported in previous gabapentin trials, suggesting better tolerability of Gralise compared with gabapentin.

## 4. Discussion

The results of this study indicate that Gralise is well tolerated and is efficacious in reducing pain intensity and the impact of pain on daily activities in patients with moderate-to-severe PMPS. Gralise therapy is also associated with a significant positive change in stress, sleep, and coping behavior. However, Gralise does not affect mood and psychophysical phenotypes in patients with PMPS.

Gabapentin is a structural analog of gamma-aminobutyric acid that binds to the α(2)-δ site of voltage-dependent calcium channels and modulates the influx of calcium, with a resulting reduction in excitatory neurotransmitter release. Gabapentin (Neurontin, FDA approved in 2002, or Horizant, FDA approved in 2011) has been used for the management of pain related to postherpetic neuralgia^19^ and moderate-to-severe primary restless legs syndrome^14^ for many years. Off-label usage for other neuropathic pain etiologies is a routine practice, despite limited clinical data beyond postherpetic neuralgia. Some of the alternative uses for gabapentin and its preparations include the minimization of lower back pain, diabetic peripheral neuropathy, neuropathic pain due to malignancies, spinal cord injuries and poststroke pain, phantom pain, HIV-associated neuropathic pain states, and trigeminal neuralgia.^8^ Gabapentin must be given at least 3 times per day, due to its short half-life, resulting in demonstrable fluctuations in plasma levels.^4^ Although effective for pain reduction, gabapentin has dose-limiting side effects that prevent some patients from achieving therapeutic plasma levels, such as somnolence, dizziness, and ataxia.

Gastroretentive gabapentin, Gralise, is a unique extended-release, once-daily formulation of gabapentin that provides both efficacy and increased tolerability. It was developed using AcuForm technology, a polymer-based drug delivery system that retains the tablet in the stomach and upper gastrointestinal tract for a sustained period. When administered with a meal, the tablet swells and remains in the stomach for up to 15 hours, releasing the drug gradually for absorption by the small intestine. The starting dose is typically 300 mg/d once daily and increased over 2 weeks to a target dose of 1800 mg/d. When administered with an evening meal, the peak dose occurs in the early morning (approximately 3 am), when patients are sleeping. Consequently, patients report a lower rate of dizziness and sedation relative to immediate release of gabapentin and pregabalin.^19^ Indeed, fatigue and dizziness observed in our study had mild symptoms, resolved spontaneously by week 7 of treatment, and were not accompanied by other AEs commonly observed in gabapentin trials, such as diarrhea, vomiting, nausea, or weight gain. Therefore, this pilot study indicates better tolerability of Gralise compared with nongastroretentive gabapentin that should be confirmed in follow-up studies.

Recently, a study in spinal stenosis patients with radicular symptoms demonstrated moderate efficacy and tolerability of Gralise.^27^ A 4-week treatment with Gralise showed a significant improvement in pain symptoms, activity limitations, emotions, and overall quality of life. Moreover, the study demonstrated improved nightly sleep and a reduction in opioid medication usage over the course of the 1 month treatment period. A majority of patients who completed the study noted a marked therapeutic effect with no side effects, suggesting that Gralise may be a safe and effective treatment option for patients with pain associated with spinal stenosis.

Here, we present that after 8 weeks of treatment with Gralise, patients showed a significant decrease not only in chronic pain severity but also in the level of stress, sleep disturbance, and pain catastrophizing behavior. Our data are in concordance with Kaye et al.^27^ and Beal et al.,^4^ both of whom suggest that Gralise positively affects pain-related psychosocial traits through pathophysiological mechanisms present in multiple neuropathic pain conditions. Pain catastrophizing, a negative coping skill in which patients have an exaggerated mental set associated with actual or anticipated pain, is a negative predictor of pain-related outcomes.^21,37^ It is common for patients with pain catastrophizing to have psychiatric comorbidities such as anxiety and depression, and not surprisingly, neuroimaging studies have found brain regions associated with emotions and pain processing to be correlated with pain catastrophizing.^35^ The mechanism by which Gralise can reduce catastrophizing behavior is unknown but may be related to the off-label role of its related compound, gabapentin, in reducing anxiety.^1^ Gabapentin does not bind directly to GABA receptors, as do the benzodiazepine and barbiturate anxiolytics, but it is thought to increase synthesis of GABA via modulation of glutamate decarboxylase and branched chain aminotransferase.^36^ It is unclear, however, why Gralise did not affect anxiety symptoms also measured in this study. This observation should be further elaborated in the next trial.

Breast cancer is the most common cancer in women worldwide. Partial or total mastectomy is the most common treatment choice in patients with breast cancer. Furthermore, with recent advances in breast cancer risk assessment and prediction, bilateral prophylactic mastectomy has become the most common risk-reducing surgery in high-risk women. Therefore, the rates of preventive mastectomy are on the rise.^39^ Almost half of patients who underwent a mastectomy report persistent postsurgical pain at least 6 months after mastectomy that can last for many years. Treatment of chronic postmastectomy pain of neuropathic pain such as PMPS is challenging due to incomplete understanding of underlying mechanisms, comorbidities, and patients heterogeneity. Perioperative use of gabapentinoid agents was found to reduce early postoperative pain.^33^ Recently, Clarke et al.^13^ conducted a meta-analysis evaluating the combined trials of both pregabalin and gabapentin to prevent chronic postsurgical pain. Although better designed and appropriately powered clinical trials are needed, the combined data support the view that perioperative administration of gabapentinoids is effective in reducing chronic postsurgical pain, including postmastectomy pain. The role for Gralise, or a drug with similar pharmacologic properties, in relief of neuropathic pain following breast surgeries including PMPS requires further investigation. Our proof-of-concept study was designed to provide feasibility data and inform study design for future double-blind randomized clinical trial in this target population. Our study had several limitations including a small sample size and lack of placebo control. While the observed reduction in pain intensity is not plausible due to natural course because PMPS in study participants persisted since breast surgery and did not decrease significantly over several years, we cannot rule out some hidden placebo effect influencing the results of this trial. Our study was enriched, as patients who previously had not tolerated gabapentin or pregabalin were excluded. Future studies with similar phenotyping approach in a large sample stratified by the type of surgery and type of pain and controlled with placebo or/and nongastroretentive drug group may elaborate on the efficacy of Gralise in treating PMPS. Including brief diaries with pain and pain-related traits measuring scales may be useful for daily assessments in addition to weekly phone interviews. Furthermore, the addition of pain screening scales such as Self-Report Leeds Assessment of Neuropathic Symptoms and Signs pain scale (S-LANSS) with a threshold score for inclusion in the study^41^ would facilitate the recruitment of patients with a neuropathic component to their postmastectomy pain and further enrich the study population. Finally, evaluation of long-term effects of Gralise (beyond 8 weeks of treatment) in the future prospective studies will help to determine its ultimate value for pain management in patients with persistent and severe PMPS.

### 4.1. Conclusions

This study, for the first time, found that an 8-week treatment with 1800 mg of Gralise significantly decreased the severity of PMPS and improved patients' sleep and catastrophizing traits associated with PMPS. The positive findings from this study suggest that Gralise may expand treatment options for PMPS; however, the possibility that pain reduction experienced by patients is, in part, due to a placebo response cannot be ruled out. Future studies would require a placebo control and use of a noninferiority trial with gabapentin. Data from this study may lead to large-scale trials on the efficacy of Gralise in comparison with other pain medicine for chronic neuropathic pain relief and functional improvement in breast cancer survivors.

## Disclosures

The authors have no conflicts of interest to declare.

This study was supported by the Investigator-Initiated Research grant (I#0042195, Depomed), internal funds of the Department of Anesthesiology, and the Clinical and Translational Science Institute (CTSI) at the University of Pittsburgh, Pittsburgh, PA.

## Appendix A Supplemental Digital Content

Supplemental Digital Content associated with this article can be found online at http://links.lww.com/PR9/A4.

## Supplementary Material

SUPPLEMENTARY MATERIAL
