# Supplementary figures and images for: Effect of gastroretentive gabapentin (Gralise) on postmastectomy pain syndrome: a proof-of-principle open-label study
Source: Pain Rep. 2017 Apr 11;2(3):e596. doi: 10.1097/PR9.0000000000000596 (PMC5741302; doi:10.1097/PR9.0000000000000596)

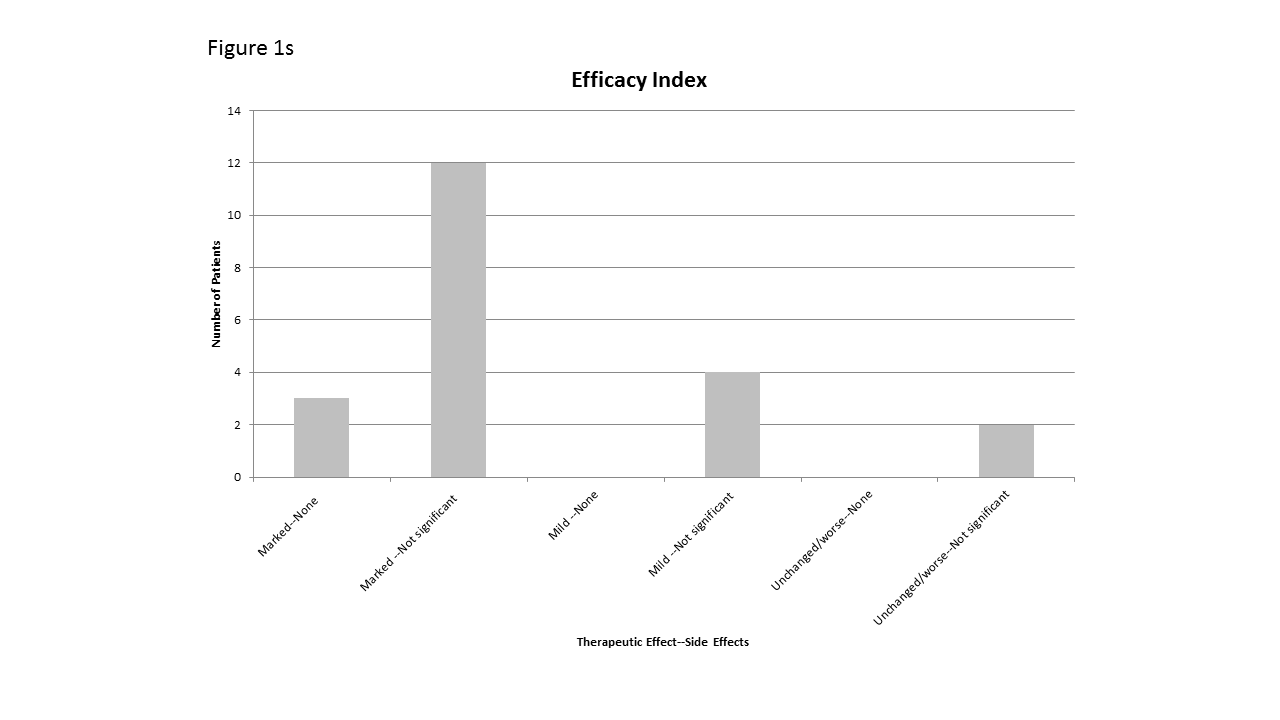

Supplement: SUPPLEMENTARY MATERIAL [file painreports-2-e596-s001.tif]

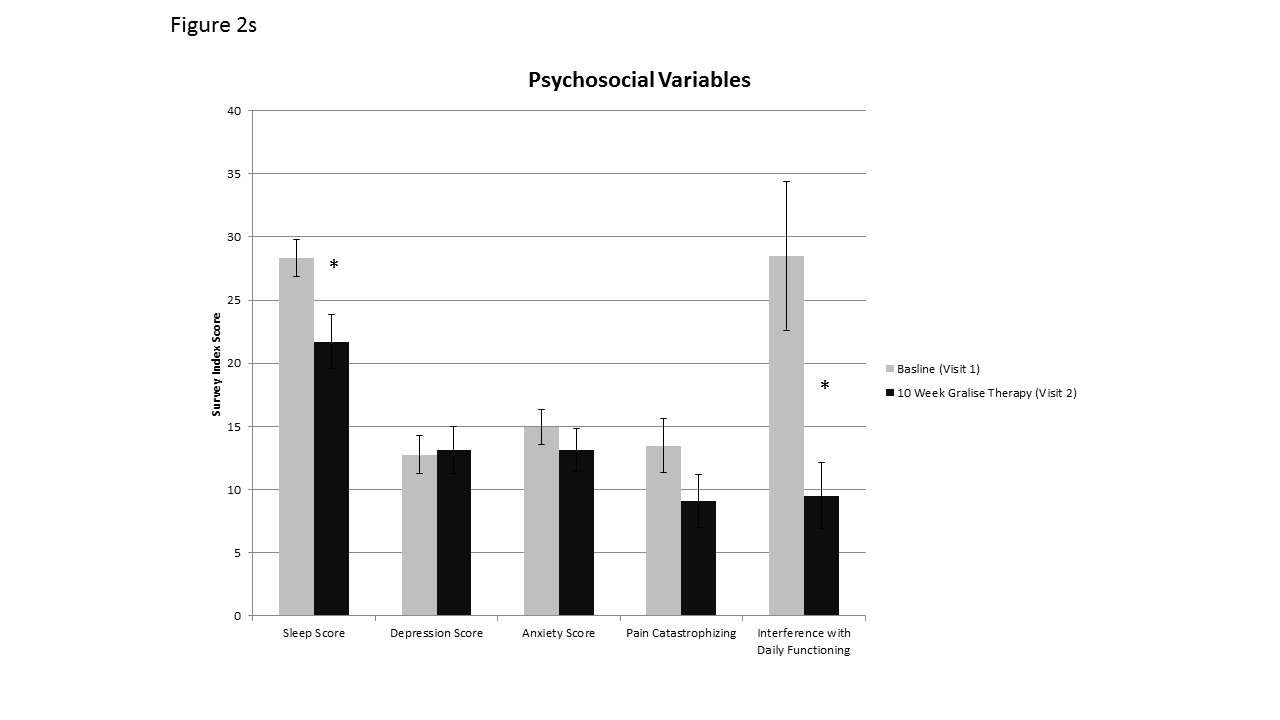

Supplement: SUPPLEMENTARY MATERIAL [file painreports-2-e596-s002.tif]
