# Supplementary material for: Effect of gastroretentive gabapentin (Gralise) on postmastectomy pain syndrome: a proof-of-principle open-label study
Source: Pain Rep. 2017 Apr 11;2(3):e596. doi: 10.1097/PR9.0000000000000596 (PMC5741302; doi:10.1097/PR9.0000000000000596)
Supplement: SUPPLEMENTARY MATERIAL [file painreports-2-e596-s003.docx]

Table 1s Study schedule/procedures

| **Protocol Activity** | **Screening** | **Pre-treatment** | **Treatment** | **Post-treatment** | **Follow-up** |
| --- | --- | --- | --- | --- | --- |
| **Study week** | **-4 - 0** | **1-2** | **3-10** | **11** | **12** |
| **Clinical visit** | **Visit 1 (week 0, baseline)** |  | **Visit 2 (week 10)** |  |  |
| **Phone visit** | **Week 0** | **Week 1** | **weekly** |  | **Week 12** |
| **Informed Consent** | X |  |  |  |  |
| **Medical History & Demographics** | X |  |  |  |  |
| **Physical Exam** | X |  |  |  |  |
| **Inclusion/exclusion criteria** | X |  |  |  |  |
| **Vital signs** | X |  | X X |  |  |
| **Blood collection** | X |  |  |  |  |
| **BPI** | X | X | X |  |  |
| **PROMIS questionnaires** | X |  | X |  |  |
| **QST session** | X |  | X |  |  |
| **AE recording** |  | X | X X | X | X |
